# *MDR* 1 activation is the predominant resistance mechanism selected by vinblastine in MES-SA cells

**DOI:** 10.1054/bjoc.2000.1371

**Published:** 2000-09-04

**Authors:** G K Chen, G E Durán, A Mangili, L Beketic-Oreskovic, B I Sikic

**Affiliations:** Oncology Division, Department of Medicine, and the Cancer Biology Program, Stanford University School of Medicine, Stanford, CA 94305–5151, USA

**Keywords:** multidrug resistance, P-glycoprotein, vinblastine, fluctuation analysis

## Abstract

Single-step selection with vinblastine was performed in populations of the human sarcoma cell line MES-SA, to assess cellular mechanisms of resistance to the drug and mutation rates via fluctuation analysis. At a stringent selection with 20 nM vinblastine, resulting in 5–6 logs of cell killing, the mutation rate was 7 × 10^–7^per cell generation. Analysis of variance supported the hypothesis of spontaneous mutations conferring vinblastine resistance, rather than induction of adaptive response elements. Surviving clones displayed a stable multidrug resistance phenotype over a 3-month period. All propagated clones demonstrated high levels of resistance to vinblastine and paclitaxel, and lower cross-resistance to doxorubicin and etoposide. Activation of *MDR* 1 gene expression and P-glycoprotein function was demonstrable in all clones. No elevation was found in the expression of the *mrp* gene, the LRP-56 major vault protein and β-tubulin isotypes (M40, β4, 5β, and β9) in these mutants. We conclude that initial-step resistant mechanism in these vinblastine-selected mutants commonly arises from a stochastic mutation event with activation of the *MDR* 1 gene. © 2000 Cancer Research Campaign


*British Journal of Cancer* (2000) **83**(7), 892–898
© 2000 Cancer Research Campaign
doi: 10.1054/ bjoc.2000.1371, available online at http://www.idealibrary.com on IDEAL®

### *MDR*1 activation is the predominant resistance mechanism selected by vinblastine in MES-SA cells

**GK Chen, GE Durán, A Mangili, L Beketic-Oreskovic and BI Sikic**

Oncology Division, Department of Medicine, and the Cancer Biology Program, Stanford University School of Medicine, Stanford, CA 94305–5151, USA

**Summary** Single-step selection with vinblastine was performed in populations of the human sarcoma cell line MES-SA, to assess cellular mechanisms of resistance to the drug and mutation rates via fluctuation analysis. At a stringent selection with 20 nM vinblastine, resulting in 5–6 logs of cell killing, the mutation rate was $7 \times 10^{-7}$ per cell generation. Analysis of variance supported the hypothesis of spontaneous mutations conferring vinblastine resistance, rather than induction of adaptive response elements. Surviving clones displayed a stable multidrug resistance phenotype over a 3-month period. All propagated clones demonstrated high levels of resistance to vinblastine and paclitaxel, and lower cross-resistance to doxorubicin and etoposide. Activation of *MDR*1 gene expression and P-glycoprotein function was demonstrable in all clones. No elevation was found in the expression of the *mrp* gene, the LRP-56 major vault protein and β-tubulin isotypes (M40, β4, 5β, and β9) in these mutants. We conclude that initial-step resistant mechanism in these vinblastine-selected mutants commonly arises from a stochastic mutation event with activation of the *MDR*1 gene. © 2000 Cancer Research Campaign

**Keywords**: multidrug resistance; P-glycoprotein; vinblastine; fluctuation analysis

P-glycoprotein (P-gp) is a multidrug efflux pump encoded by the *MDR*1 gene. P-gp is expressed in some normal and malignant tissues, and associated with clinical multidrug resistance (MDR) and poor prognosis in many cancer types (Ling, 1992; Gottesman, 1993; Sikic et al, 1997). Clinical trials using P-gp inhibitors such as the cyclosporin analogue valspodar (PSC 833) combined with chemotherapeutic agents are currently being carried out to reverse or prevent clinical drug resistance (Fisher and Sikic, 1995).

*Vinca* alkaloids such as vinblastine are potent anti-tumour agents and widely used in the chemotherapy of cancers. Cellular mechanisms of resistance to *vinca* alkaloids include expression of *MDR*1/P-gp, the *mrp* gene, alterations in tubulin or microtubule-associated proteins, and alterations in the regulation of programmed cell death or apoptosis (Cabral and Barlow, 1989; Haber et al, 1995; Giannakakou et al, 1997; Dumontet and Sikic, 1999). Most cellular models of resistance to vinblastine were established by step-wise selection of cancer cells up to very high concentrations. Single-step selection and fluctuation analysis provide a useful genetic tool to investigate the frequency and the nature of various resistance mechanisms to cytotoxic agents (Luria and Delbrück, 1943; Goldie and Coldman, 1979; Kendal and Frost, 1988; Chen et al, 1994; Jaffrézou et al, 1994; Beketic-Oreskovic et al, 1995; Dumontet et al, 1996).

In previous reports, we have identified the stochastic nature of initial resistance mechanisms to etoposide, doxorubicin, paclitaxel, and doxorubicin in the presence of valspodar (PSC 833), in the human sarcoma cell line MES-SA (Jaffrézou et al, 1994; Chen et al, 1994; Beketic-Oreskovic et al, 1995; Dumontet et al, 1996). In this study, we characterized the rate and mechanisms of resistant cells derived from vinblastine single-step selection. Acquired *MDR*1 expression in these single-step mutants was found in all resistant clones and was associated with the initial resistant phenotype in these clones.

*Received 4 August 1999*
*Revised 31 May 2000*
*Accepted 4 June 2000*

*Correspondence to:* BI Sikic

### MATERIALS AND METHODS

### Drugs and chemicals

Paclitaxel, etoposide, vinblastine, and doxorubicin were obtained from the drug repository of the National Cancer Institute, NIH (Bethesda, MD, USA). Valspodar (PSC 833) was a gift from Novartis Pharmaceuticals (East Hanover, NJ, USA). [$^3$H]-Paclitaxel and [$^3$H]-vinblastine were purchased from Amersham Life Science Corporation (Arlington Heights, IL, USA). Other chemicals were purchased from Sigma Chemical Company (St Louis, MO, USA).

### Cells and cell culture

The development, characterization, and culture of the human uterine sarcoma cell line MES-SA and its multistep-selected MDR variant Dx5 cells have been described (Harker et al, 1983; Harker and Sikic, 1985). MES-SA cells have a pseudodiploid karyotype of 45XX, which has remained stable over a 17-year period of cultivation. Monolayer cultures of MES-SA cells are not growth limited at low cell densities and have a plating efficiency greater than 85% (Chen GK and Sikic BI, unpublished data). Therefore, these human sarcoma cells are especially useful for fluctuation analysis.

### Luria–Delbrück fluctuation analysis

Subclonal MES-SA cells were expanded for fluctuation analysis. Eight 25 cm$^2$ tissue culture flasks (Corning Glass Works, Corning,

NY, USA) were seeded with MES-SA cells at a low density (1000 cells per flask) and allowed to grow to near confluence ($2 \times 10^6$ cells). The cells from each flask were seeded into separate 96-well plates ($1.7 \times 10^6$ cells per plate). After 4 h of incubation to ensure active growth, cells were treated with 20 nM vinblastine. Preliminary experiments had demonstrated that this concentration of vinblastine resulted in approximately 5 logs of cell killing in MES-SA cells. The drug-containing medium was changed every other day for 14 days, and then replaced by drug-free medium. Surviving colonies were allowed to grow for another 2 weeks and were then individually harvested and propagated in drug-free medium for further studies. The mutation rate was calculated according to the method of Catcheside (Catcheside, 1951), from the equation:

$$\mu = 2 \ln 2(r_2/N_2 - r_1/N_1)/g$$

where $\mu$ is the mutation rate per cell generation, r represents the numbers of resistant colonies at times 1 and 2, N is the initial cell number adjusted for plating efficiency, and g is the number of cell generations.

In a control experiment, bulk populations of MES-SA cells without expansion of the populations prior to drug exposure were treated directly with vinblastine. In this experiment, six 96-well plates were seeded with $1.7 \times 10^6$ cells per plate from a single population of $1.0 \times 10^7$ cells and received identical exposure to vinblastine as above.

### Cytotoxicity assays and modulation of MDR by P-gp inhibitors

Cytotoxicity assays and assessment of reversal of the MDR phenotype were performed using the 72 h MTT [3-(4,5-dimethylthiazol-2-yl)-2,5-diphenyltetrazolium bromide] colourimetric assay as previously described (Chen et al, 1994). The $IC_{50}$ (drug concentration resulting in 50% inhibition of MTT dye formation compared with the controls) was determined directly from semilogarithmic dose–response curves.

### Cell growth rate

The doubling times of MES-SA and the isolated clones were determined by seeding cells in 96-well plates and incubating at 37°C for 4, 24, 48, and 72 h. The cellular growth rate was estimated by the MTT colourimetric method. Population doubling time (DTs) was calculated as previously described (Chen et al, 1994).

### Flow cytometric analysis of P-gp expression

Retention of rhodamine 123 (Rh-123) was determined by a FACS-II™ dual laser flow cytometer (Becton-Dickinson Corp, Mountain View, CA, USA). Double labeling was performed with Rh-123 and the monoclonal antibody UIC2 (Immunotech Corp, Westbrook, ME, USA) (Chen et al, 1994). Cells were incubated with or without 2 μM valspodar at 37°C, and stained with Rh-123 (0.1 μg $ml^{-1}$) for 45 min, followed by 20 min drug efflux in the presence or absence of 2 μM valspodar.

### Western blotting and immunohistochemistry

The light-enhanced chemiluminescence Western blot protocol (Amersham Life Science) was used for the detection of P-gp. Total cell lysates were used for P-gp immunoblotting with the monoclonal antibody C219 (Signet Inc, Dedham, MA, USA) (Chen et al, 1994; Beketic-Oreskovic et al, 1995). P-gp and LRP (p110) expression were also assessed by immunocytochemistry, utilizing the UIC2 and LRP-56 monoclonal antibodies (Caltag Laboratories Inc, San Francisco, CA, USA) (Chen et al, 1994; Beketic-Oreskovic et al, 1995).

### Drug accumulation

Intracellular [$^3$H]-vinblastine, [$^3$H]-paclitaxel, [$^3$H]-daunorubicin, and [$^3$H]-etoposide accumulations in the presence or absence of the P-gp inhibitors valspodar and verapamil were quantitated using radiolabeled drugs, and normalized to whole cellular protein content (Beketic-Oreskovic et al, 1995; Chen et al, 1997).

### Amplimers and RT-PCR

The amplimers and methodology for RT-PCR of *MDR*1, *mrp*, β-tubulin isotypes, and rRNA have been previously documented (Beketic-Oreskovic et al, 1995; Dumontet et al, 1996; Chen et al, 1997).

### RESULTS

### Fluctuation analysis and isolation of vinblastine-resistant mutants

Table 1 presents the results of the fluctuation analysis experiment in which populations of MES-SA sarcoma cells were selected with vinblastine in a single step. The mean number of colonies surviving drug exposure in the fluctuation test group was 14 per cell population, each propagated from an initial seeding of $10^3$ drug-sensitive cells, with a statistical variance of 107 from the mean. In contrast, the mean number of colonies surviving drug exposure among the control populations (derived from bulk cultures of cells) was 16 per population, with a variance of 37 (Table 1). The larger ratio of variance relative to the mean number of survivors in the fluctuation group (7.6 vs 2.3) supports the hypothesis of spontaneous mutations conferring resistance to vinblastine in these cells, rather than an effect of induction of resistance by drug exposure. The mutation rate for vinblastine resistance in this single-step selection was estimated to be $7 \times 10^{-7}$ per cell per generation according to the method of Catcheside (1951) (Table 1).

### MDR phenotype and its reversal

A total of 13 clones which survived vinblastine exposure were isolated, propagated and tested for resistance to the selecting agent as well as to doxorubicin, paclitaxel and etoposide. These clones included at least one from each of the eight original populations. There was no detectable alteration in proliferation rates among these clones compared to the parental cell line, which has a generation time of approximately 22 h (data not shown). All of the propagated clones demonstrated an MDR phenotype (Table 2), typical of *MDR*1 expression. Thus, all clones showed marked cross-resistance to paclitaxel (ranging from 50- to 200-fold). Cross-resistance to paclitaxel was usually greater than to the selecting agent vinblastine, and lower to doxorubicin and etoposide (Table 2). Five other clones were propagated (VL20-1.2, VL20-4.2,

© 2000 Cancer Research Campaign

**Table 1** Fluctuation analysis of vinblastine-resistant MES-SA variants (14 days exposure in 20 nM vinblastine)

| Plate | Fluctuation group[a] | | Control group | |
|---|---|---|---|---|
| | Total colonies per plate | Wells containing colonies per plate | Total colonies per plate | Wells containing colonies per plate |
| 1 | 5 | 4 | 21 | 10 |
| 2 | 6 | 3 | 23 | 16 |
| 3 | 3 | 2 | 20 | 19 |
| 4 | 24 | 18 | 11 | 11 |
| 5 | 19 | 16 | 14 | 15 |
| 6 | 31 | 24 | 8 | 8 |
| 7 | 6 | 4 | – | – |
| 8 | 16 | 10 | – | – |
| Total | 110 | 81 | 97 | 79 |
| Mean | 14 | 10 | 16 | 13 |
| Variance | 107 | 69 | 37 | 17 |
| Mutation rate[b] | $7.0 \times 10^{-7}$ | | | |

[a]The difference in the two groups was that a small number of cells ($10^3$) were seeded and grown separately in the fluctuation group, according to Luria and Delbrück (1943), whereas the controls utilized bulk populations of cells ($1.7 \times 10^6$) from mass cultures, prior to selection with 20 nM vinblastine; [b]calculated according to Catcheside (1951).

**Table 2** Drug sensitivity phenotype of vinblastine-resistant MES-SA variants (fold resistance[a])

| Clones | Vinblastine | Paclitaxel | Doxorubicin | Etoposide |
|---|---|---|---|---|
| *Controls* | | | | |
| MES-SA | 1 | 1 | 1 | 1 |
| D×5 | 243 | 375 | 80 | 30 |
| *Single-step mutants* | | | | |
| VL20-1.1[b] | 13 | 65 | 6 | 6 |
| VL20-2.1 | 17 | 71 | 12 | 6 |
| VL20-3.1 | 14 | 125 | 14 | 2 |
| VL20-4.1 | 70 | 172 | 38 | 5 |
| VL20-5.1 | 18 | 200 | 4 | 2 |
| VL20-6.1 | 54 | 53 | 17 | 9 |
| VL20-7.1 | 144 | 167 | 4 | 4 |
| VL20-8.1 | 36 | 50 | 10 | 3 |

[a]Fold resistance was the ratio of the $IC_{50}$s of the resistant cells to the parental MES-SA cells. The $IC_{50}$s were determined by the MTT cytotoxicity assay; [b]nomenclature of clones: e.g. VL20-1.1: VL = vinblastine; 20 = 20 nM; and 1.1 = clone number 1 isolated from population number 1 of the fluctuation analysis experiment. Each of the clones was propagated from a different population.

VL20-5.2, VL20-6.2, and VL20-8.2) and shown to have a similar drug resistance phenotype to that of their related clones in Table 2 (data not shown). The resistance to vinblastine and paclitaxel in all propagated clones was completely reversed by 2 μM valspodar. All propagated clones maintained stable resistance to vinblastine and paclitaxel over a 3-month period.

### Rh-123 retention and potentiation by valspodar

The intracellular accumulation of the fluorescent P-gp substrate Rh-123 was examined in eight resistant clones. All clones demonstrated significantly decreased accumulation of Rh-123 (Figure 1), with Rh-123 retention ranging from 14% to 56% compared to the parental MES-SA cells (100%). Valspodar at 2 μM restored the intracellular accumulation of Rh-123 to levels similar to the parental cells, which do not express P-gp (Figure 1).

### Accumulation of [$^3$H]-vinblastine, [$^3$H]-paclitaxel, and [$^3$H]-etoposide

The vinblastine selected clones demonstrated markedly reduced intracellular accumulation of [$^3$H]-vinblastine and [$^3$H]-paclitaxel relative to the wild-type MES-SA cells. Furthermore, this decreased drug accumulation was reversed by the MDR modulators valspodar and verapamil. In these experiments, 2 μM valspodar completely sensitized the vinblastine-resistant phenotype to the parental MES-SA cell level (Figure 2). Valspodar at 2 μM was more effective as a modulator in these cells than 6 μM verapamil, which only partially reversed the accumulation defects for both vinblastine and paclitaxel (Figure 3). One clone showed resistance to modulation by verapamil of the accumulation of vinblastine and paclitaxel (Figure 3). In general, these results suggest that a typical drug efflux mechanism associated with reduced accumulation of total intracellular drugs was responsible for cellular resistance to vinblastine in these clones.

### Expression of *MDR1* mRNA and P-gp

All examined clones expressed *MDR*1 mRNA transcripts and functional P-glycoprotein, compared to the parental MES-SA cells which do not express *MDR*1 (Figure 5A). P-gp expression and function were analysed by double labeling of cells using the monoclonal antibody UIC2 combined with Rh-123 accumulation. Figure 4 demonstrates the association between P-gp expression

© 2000 Cancer Research Campaign

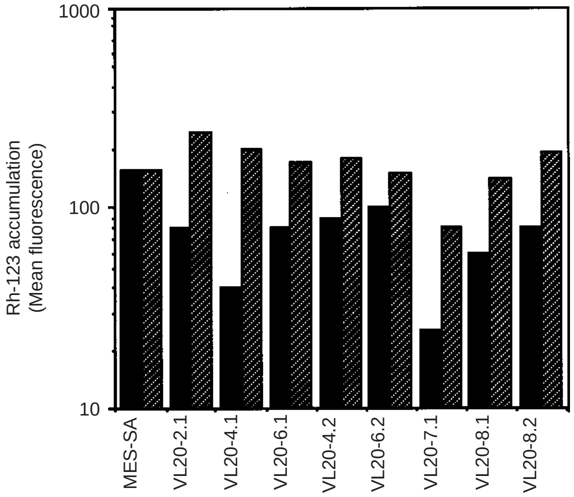

**Figure 1** Histogram of rhodamine-123 retention in clones of MES-SA cells resistant to vinblastine. Rhodamine-123 (Rh-123) retention was assayed by flow cytometry in MES-SA cells and vinblastine-resistant clones, with (▨) or without (■) 2 µM valspodar

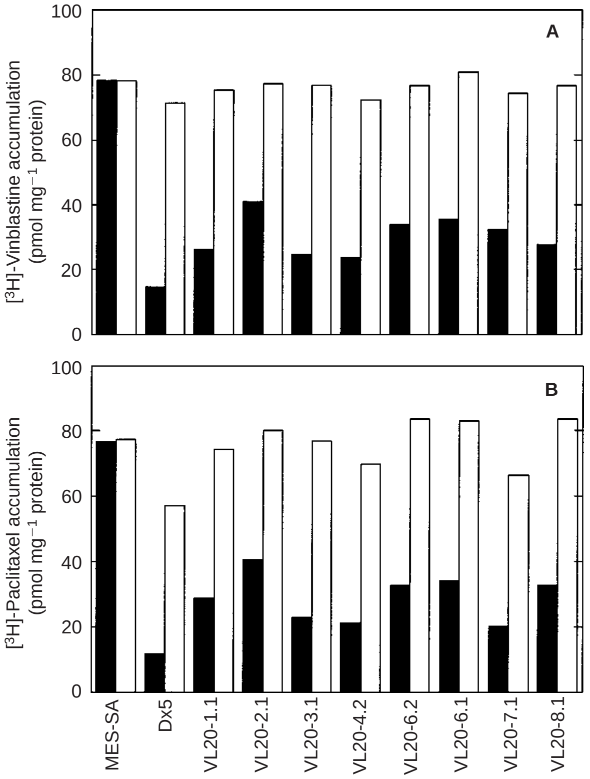

**Figure 2** The effect of 2 µM valspodar on the accumulation of [$^3$H]-vinblastine (**A**) and [$^3$H]-paclitaxel (**B**) in vinblastine-resistant clones of MES-SA cells. The final concentration of both [$^3$H]-vinblastine and [$^3$H]-paclitaxel was 40 nM. ■ = without valspodar; □ = with valspodar

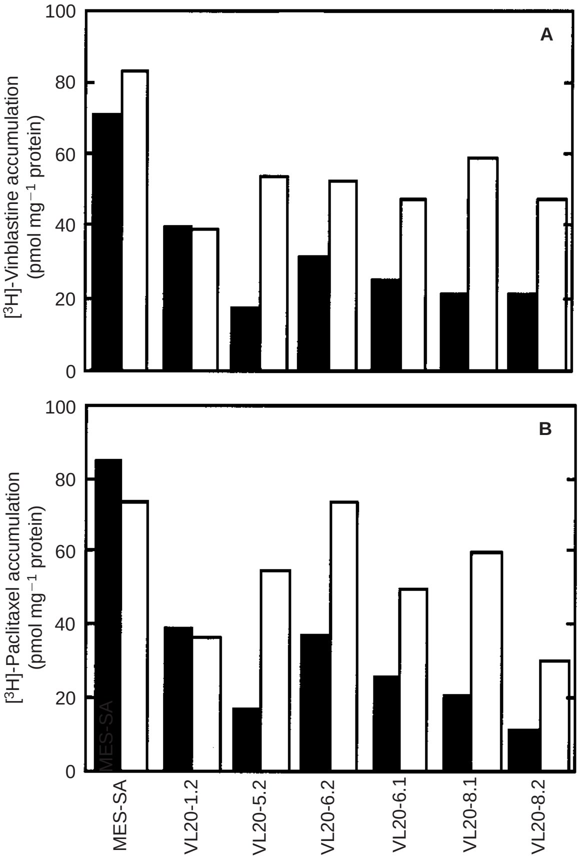

**Figure 3** The effect of 6 µM verapamil on the accumulation of [$^3$H]-vinblastine and [$^3$H]-paclitaxel in vinblastine-resistant clones of MES-SA cells. The final concentration of both [$^3$H]-vinblastine and [$^3$H]-paclitaxel was 40 nM. ■ = without verapamil; □ = with 6 µM verapamil and decreased retention of Rh-123 in the resistant clones compared to parental MES-SA cells. There was a highly significant correlation of P-gp/UIC2 expression with decreased Rh-123 accumulation among the nine tested clones (Figure 4D, $r = 0.84$, $P < 0.002$). Expression of P-gp was confirmed in these clones by Western blotting using the monoclonal antibody C219 (Figure 5B).

### LRP and *mrp* expression

Expression of the major vault protein LRP was assessed by immunocytochemistry, utilizing the LRP-56 monoclonal antibody. The parental, drug-sensitive MES-SA cells stained positively for LRP (p110). Vinblastine-selected variants had a similar degree of LRP-56 staining compared to parental MES-SA cells (data not shown). None of the vinblastine-selected clones had an increase in *mrp* expression (Figure 6) or altered β-tubulin isotypes (M40, β4, 5β, and β9), assessed by RT-PCR (data not shown).

### DISCUSSION

Fluctuation analysis provides a powerful approach to studying the nature and rate of acquired resistance to anticancer agents, although such studies are difficult and laborious in mammalian cells (Luria and Delbrück, 1943; Goldie and Coldman, 1979; Kendal and Frost, 1988; Chen et al, 1994; Jaffrézou et al, 1994; Beketic-Oreskovic et al, 1995; Dumontet et al, 1996). The human sarcoma cell line MES-SA is particularly suitable for such studies because of its high plating efficiency, relative drug sensitivity, and karyotypic and genetic stability over two decades of growth (Harker et al, 1983; Chen and Sikic, unpublished data). Previously,

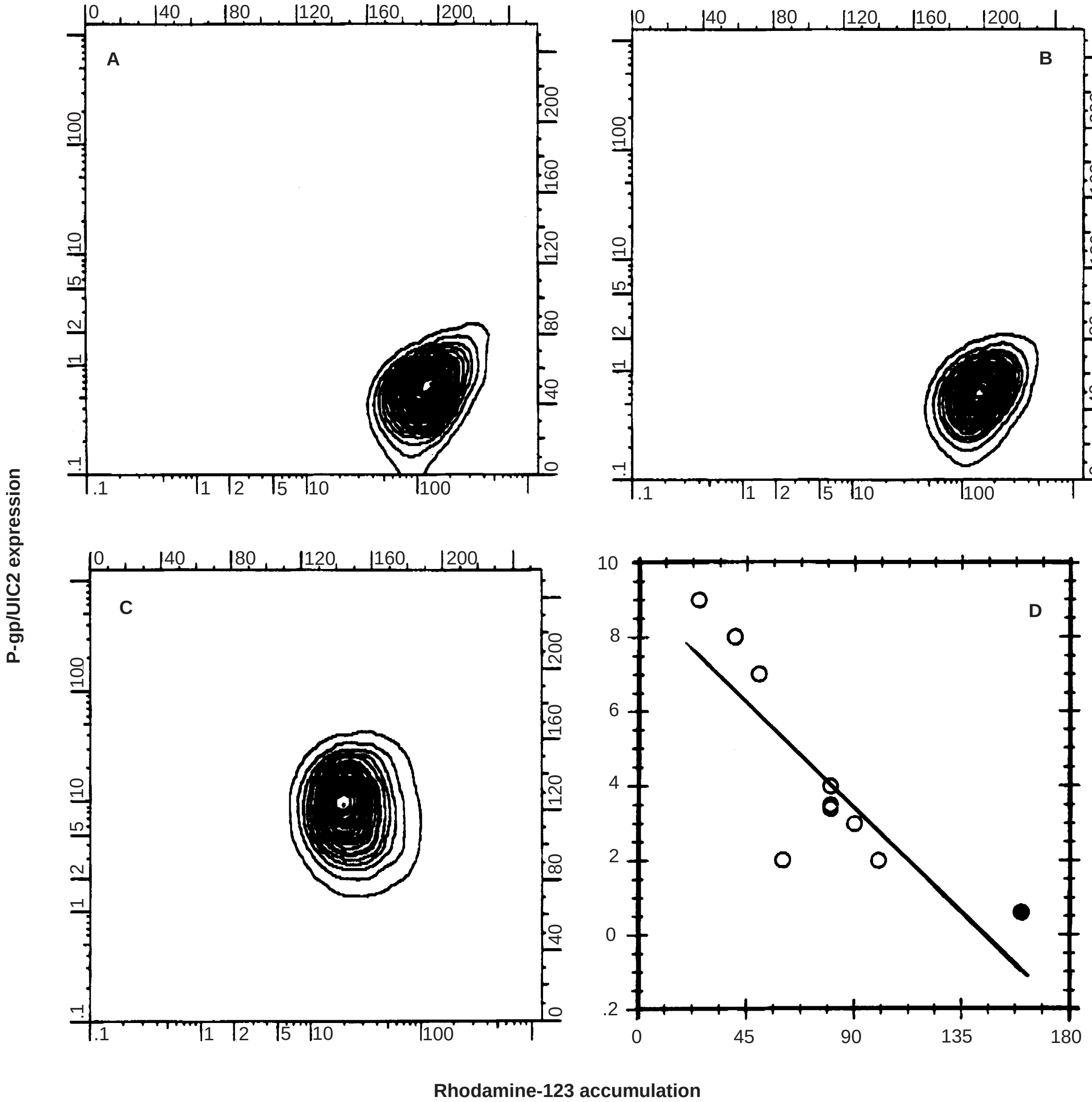

**Figure 4** Flow cytometric analysis of rhodamine-123 retention and P-gp expression in MES-SA and vinblastine-selected mutants. Contour intensity maps (5% probability plots) of the cells were obtained by two-colour flow cytometry using rhodamine-123 (x-axis) and the UIC2 monoclonal antibody for P-gp (y-axis). An IgG2a isotype monoclonal antibody was used as an isotype control. (**A**) MES-SA cells stained with an IgG2a monoclonal antibody control; (**B**) MES-SA cells stained with the UIC2 monoclonal antibody; (**C**) Clone VL20–7.1, stained with UIC2, representative of eight vinblastine-selected clones; (**D**) Correlation of rhodamine-123 retention and P-gp expression by flow cytometry in clones of MES-SA cells resistant to vinblastine (○). The linear correlation between Rh-123 accumulation and P-gp/UIC2 expression among various clones selected by vinblastine exposure is highly significant ($r = 0.84$, $P < 0.002$). The black dot (•) represents the parental MES-SA cells we have shown that activation of the *MDR*1 gene in MES-SA sarcoma cells could be acquired by stochastic genetic events (Chen et al, 1994; Dumontet et al, 1996). *MDR*1 activation was present in all 13 mutants selected with doxorubicin (Chen et al, 1994), and in four of nine (44%) selected with paclitaxel (Dumontet et al, 1996).

It has been shown that multiple mechanisms are involved in cellular resistance to *vinca* alkaloids (Cabral and Barlow, 1989; Haber et al, 1995; Giannakakou et al, 1997; Dumontet and Sikic, 1999). In order to investigate the nature or origin of acquired resistance to *vinca* alkaloids in MES-SA cells, we used fluctuation analysis to examine the potential diversity of resistance mechanisms that may arise among eight parallel cell populations in a single-step exposure to vinblastine. We found that all eight mutants from separate parental cell populations acquired *MDR*1 expression and manifested the MDR phenotype characteristic of P-gp.

In theory, if resistance was acquired by induced events or directed mutations, the number of surviving colonies would be expected to have a Poisson distribution, with the variance close to the mean (Luria and Delbrück, 1943; Goldie and Coldman, 1979; Kendal and Frost, 1988). In this study, the variance in number of surviving colonies per plate was over 8-fold greater than the mean,

© 2000 Cancer Research Campaign

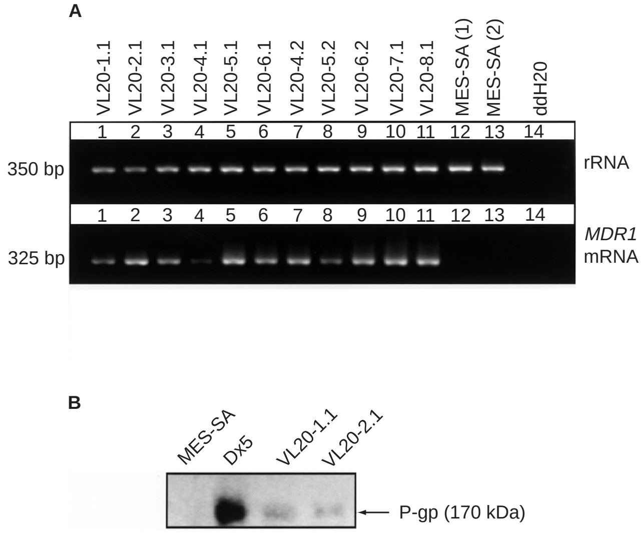

**Figure 5** (**A**) Analysis of *MDR*1 mRNA expression in vinblastine-resistant mutants using RT-PCR. Parental MES-SA cells were negative controls. TM 6, the sixth transmembrane segment of P-gp (325 bp) was amplified by a specific primer as previously described (Chen et al, 1997). (**B**) Western blotting of vinblastine single-step mutants. Total cellular lysates were separated by 8% SDS-polyacrylamide gel, and transferred onto a nitrocellulose membrane. P-gp expression was detected by ECL Western blotting using the monoclonal antibody C219 as previously described (Chen et al, 1997). MES-SA and Dx5 cells were used as negative and positive controls respectively. Two representative vinblastine-selected clones are shown (VL20–1.1 and VL20–2.1).

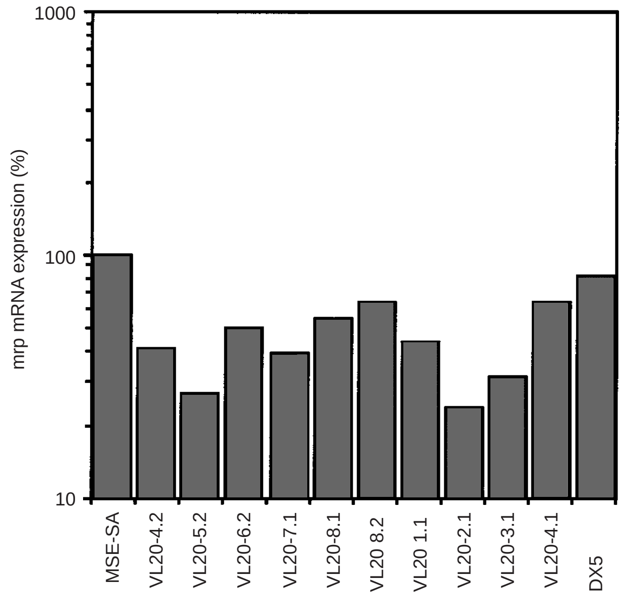

**Figure 6** Histogram of *mrp* mRNA levels in vinblastine-selected MDR mutants. *Mrp* mRNA expression in vinblastine-selected mutants was determined by RT-PCR, *r*RNA was used as the control gene to normalize expression as described. MES-SA and its MDR subline were used a controls.

indicating a spontaneous event rather than direct induction of *MDR*1 expression by drug exposure. These data are strong evidence that a random mutational mechanism caused the cellular resistance to vinblastine. The Luria–Delbrück test may be particularly applicable for analysis of mutations due to single-step irreversible events, rather than two- or multiple-step genetic changes such as gene amplification (Kimmel and Axelrod, 1994). The estimated rate of mutation ($7 \times 10^{-7}$ per cell generation) conferring resistance to 20 nM vinblastine in MES-SA cells is similar to that which we observed for doxorubicin resistance ($1.8 \times 10^{-6}$) in the same cells, and somewhat lower than that which has been described for some gene amplification events in other cell lines, $1–7 \times 10^{-5}$ per cell generation (Tlsty et al, 1989; Baker et al, 1974; Crawford et al, 1983; Cole et al, 1976). It should be pointed out that the spontaneous mutation rate is not always constant, but can be highly dependent on the parental cell line, drug concentration, and other experimental conditions (Boesen et al, 1994).

A significant level of *MDR*1 transcripts and P-gp expression were verified in all tested clones which were initially cross-resistant to the MDR-related drugs doxorubicin, vinblastine, paclitaxel, and etoposide. There was some variability in the pattern of resistance, which may reflect altered forms or post-translational regulation of P-gp in these cells or clonal variation in other genes. In addition to ruling out alterations in LRP and *mrp* expression, we found no obvious alterations in tubulin content (data not shown) or β-tubulin isotype expression in these mutants. Functional analysis of P-gp by combined Rh-123 retention and UIC2 labeling was performed in the vinblastine-resistant clones. The clones manifested a typical *MDR*1 phenotype, including decreased intracellular rhodamine 123, [$^3$H]-paclitaxel, and [$^3$H]-vinblastine accumulations which were reversed by 2 μM valspodar (Figures 1–4; Table 2). Thus, these vinblastine-selected clones are phenotypically similar to the previously published MES-SA mutants selected by doxorubicin with activation of *MDR*1 (Chen et al, 1994). The mechanisms of acquired MDR related to activation of *MDR*1 are not well understood. The genetic mechanisms that regulate *MDR*1 expression acquired during drug selection may differ from those responsible for constitutive expression, and these single-step models may provide new insights into this question.

In conclusion, these data demonstrate that resistance to 20 nM vinblastine in the human sarcoma cell line MES-SA is not induced but arises spontaneously with an apparent mutation rate of $7 \times 10^{-7}$ per cell generation. Vinblastine exposure selected predominantly for *MDR*1-expressing variants. It is likely that these mutants arose from one major event leading to an activation of *MDR*1. The detailed nature of the *MDR*1 gene activation in these clones is being investigated.

### ACKNOWLEDGEMENTS

This work was supported by American Cancer Society grant RPG-88–005–11-CDD (BIS), NIH Grant R01 52168 (BIS), and PHS grant T32 CA 09302 (Cancer Biology Training Program).

### REFERENCES

Baker RM, Burnette DM, Mankovitz R, Thompson LH, Whitemore GF, Siminovitch L and Till JE (1974) Ouabain-resistant mutants of mouse and hamster cell in culture. *Cell* **1**: 9–21

Beketic-Oreskovic L, Durán GE, Chen G, Dumontet C and Sikic BI (1995) Decreased mutation rate for cellular resistance to doxorubicin and suppression of mdr1 gene activation by the cyclosporin PSC 833. *J Natl Cancer Inst* **87**: 1593–1602

Boesen JJ, Niericker MJ, Dieteren N and Simons JW (1994) How variable is a spontaneous mutation rate in cultured mammalian cells? *Mutat Res* **307**: 121–129

*© 2000 Cancer Research Campaign* 

Cabral F and Barlow SB (1989) Mechanisms by which mammalian cells acquire resistance to drugs that affect microtubule assembly. *FASEB J* **3**: 1593–1599
Catcheside DG (1951) *The Genetics of Micro-Organisms*, pp. 158–163. Sir Isaac Pitman & Sons Ltd: London
Chen G, Jaffrézou J-P, Fleming WH, Durán GE and Sikic BI (1994) Prevalence of multidrug resistance related to activation of the mdr1 gene in human sarcoma mutants derived by single-step doxorubicin selection. *Cancer Res* **54**: 4980–4987
Chen G, Durán GE, Steger KA, Lacayo NJ, Jaffrézou J-P, Dumontet C and Sikic BI (1997) Multidrug-resistant human sarcoma cells with a mutant P-glycoprotein, altered phenotype, and resistance to cyclosporins. *J Biol Chem* **272**: 5974–5982
Cole J, Arlett CF and Green MH (1976) The fluctuation test as a more sensitive system for determining induced mutation in L5178Y mouse lymphoma cells. *Mutat Res* **41**: 377–386
Crawford BD, Barrett JC and Ts'o OP (1983) Neoplastic conversion of proneoplastic Syrian hamster cells: rate estimation by fluctuation analysis. *Mol Cell Biol* **3**: 931–945
Dumontet C and Sikic BI (1999) Mechanisms of action of and resistance to anti-tubulin agents: microtubule dynamics, drug transport, and cell death. *J Clin Oncol* **17**: 1061–1070
Dumontet C, Durán GE, Steger KA, Beketic-Oreskovic L and Sikic BI (1996) Resistance mechanisms in human sarcoma mutants derived by single-step exposure to paclitaxel (Taxol). *Cancer Res* **56**: 1091–1097
Fisher GA and Sikic BI (1995) Clinical studies with modulators of multidrug resistance. In: *Drug Resistance in Clinical Oncology and Hematology*. Hematology/Oncology Clinics of North America, Vol. 9, No. 2. Fisher GA and Sikic BI (eds) pp 363–382. WB Saunders Co: Philadelphia
Giannakakou P, Sackett DL, Kang YK, Zhan Z, Buters JT, Fojo T and Poruchynsky MS (1997) Paclitaxel-resistant human ovarian cancer cells have mutant beta-tubulins that exhibit impaired paclitaxel-driven polymerization. *J Biol Chem* **272**: 17118–17125
Goldie JH and Coldman AJ (1979) A mathematical model for relating the drug sensitivity of tumors to the spontaneous mutation rate. *Cancer Treat Rep* **63**: 1727–1733
Gottesman MM (1993) How cancer cells evade chemotherapy: Sixteenth Richard and Hinda Rosenthal Foundation Award Lecture. *Cancer Res* **53**: 747–754
Haber M, Burkhart CA, Regl DL, Madafiglio J, Norris MD and Horwitz SB (1995) Altered expression of M beta 2, the class II beta-tubulin isotype, in a murine J774.2 cell line with a high level of taxol resistance. *J Biol Chem* **270**: 31269–31275
Harker WG and Sikic BI (1985) Multidrug (pleiotropic) resistance in doxorubicin-selected variants of the human sarcoma cell line MES-SA. *Cancer Res* **45**: 4091–4096
Harker WG, MacKintosh FR and Sikic BI (1983) Development and characterization of a human sarcoma cell line, MES-SA, sensitive to multiple drugs. *Cancer Res* **43**: 4943–4950
Jaffrézou J-P, Chen G, Durán GE, Kuhl JS and Sikic BI (1994) Mutation rates and mechanisms of resistance to etoposide determined from fluctuation analysis. *J Natl Cancer Inst* **86**: 1152–1158
Kendal WS and Frost P (1988) Pitfalls and practice of Luria–Delbrück fluctuation analysis: A review. *Cancer Res* **48**: 1060–1065
Kimmel M and Axelrod DE (1994) Fluctuation test for two-stage mutations: application to gene amplification. *Mutat Res* **306**: 45–60
Ling V (1992) P-glycoprotein and resistance to anticancer drugs. *Cancer* **69**: 2603–2609
Luria SE and Delbrück M (1943) Mutations of bacteria from virus sensitive to virus resistance. *Genetics* **28**: 491–511
Sikic BI, Fisher GA, Lum BL, Halsey J, Beketic-Oreskovic L and Chen G (1997) Modulation and prevention of multidrug resistance by inhibitors of P-glycoprotein. *Cancer Chemother Pharmacol* **40**: S13
Tlsty TD, Margolin BH and Lum K (1989) Differences in the rates of gene amplification in nontumorigenic and tumorigenic cell lines as measured by Luria-Delbrück fluctuation analysis. *Proc Natl Acad Sci USA* **86**: 9441–9445

 *© 2000 Cancer Research Campaign*
